# Low-Risk Myelodysplastic Syndrome Revisited: Morphological, Autoimmune, and Molecular Features as Predictors of Outcome in a Single Center Experience

**DOI:** 10.3389/fonc.2022.795955

**Published:** 2022-03-22

**Authors:** Bruno Fattizzo, Giorgia Virginia Levati, Juri Alessandro Giannotta, Giulio Cassanello, Lilla Marcella Cro, Anna Zaninoni, Marzia Barbieri, Giorgio Alberto Croci, Nicoletta Revelli, Wilma Barcellini

**Affiliations:** ^1^ Hematology Unit, Fondazione IRCCS Ca’ Granda Ospedale Maggiore Policlinico, Milan, Italy; ^2^ Department of Oncology and Hemato-Oncology, University of Milan, Milan, Italy; ^3^ Cytofluorimetry Service, Central Laboratory Unit, Fondazione IRCCS Ca’ Granda Ospedale Maggiore Policlinico, Milan, Italy; ^4^ Pathology Unit, Fondazione IRCCS Ca’ Granda Ospedale Maggiore Policlinico, Milan, Italy; ^5^ Immunohematology Reference Laboratory, Department of Transfusion Medicine and Hematology, Fondazione IRCCS Ca’ Granda Ospedale Maggiore Policlinico, Milan, Italy

**Keywords:** low-risk myelodysplastic syndromes, autoimmunity, somatic mutations, hypoplastic myelodysplastic syndromes, bone marrow microenvironment

## Abstract

Low-risk myelodysplastic syndromes (LR-MDS) are a very heterogeneous disease, with extremely variable clinical features and outcome. Therapeutic strategies are still limited and mainly consist of erythropoiesis-stimulating agents (ESAs) and transfusion support. The contribution of molecular lesions and of autoimmune phenomena to pathogenesis and clinical course, including leukemic evolution, is a field of open investigation. We analyzed data from a cohort of 226 patients with LR-MDS followed at our center in the last 20 years, focusing on morphological, immunological (antiplatelets and anti-erythrocyte autoantibodies, anti-erythroblast antibodies), and molecular features. Hypoplastic bone marrow was found in 7% of the cases correlating with younger age, deeper cytopenia, lower dysplasia, and worse response to ESAs. A marker of autoimmunity was observed in 46% of the tested cases, who were younger, were less frequent dysplastic changes, and responded better to ESAs and steroids. Finally, 68% of the tested cases displayed at least one somatic mutation, most commonly SF3B1, TET2, ASXL1, and SRSF2, associated with older age, presence of neutropenia, and lower response to ESAs. Leukemic evolution (2.2%) was associated with presence of somatic mutations, and survival was favorably related to response to ESAs and transfusion independence. Overall, granular evaluation and re-evaluation are pivotal in LR-MDS patients to optimize clinical management.

## Introduction

Myelodysplastic syndromes (MDS) are a heterogeneous group of hematological malignant diseases characterized by ineffective hematopoiesis leading to peripheral blood cytopenias, and by a variable rate of progression to acute myeloid leukemia (AML) (1). Median age at onset is about 70 years, and overall survival varies between 8 months to 8 years depending on risk classification. The latter is essentially defined by the international prognostic scoring system (IPSS) that divided patients into “higher risk” (HR-MDS, IPSS high, or intermediate-2) and “lower risk” (LR-MDS, IPSS low, or intermediate-1), basing on the severity of cytopenias, bone marrow blasts, and chromosomal abnormalities (2). Recently, a revised IPSS version (IPSS-R) was introduced, including a refined cytogenetic classification, definition of cytopenia, and categorization of bone marrow blasts. By IPSS-R, very low and low subgroups are considered LR-MDS, while high and very high-risks HR-MDS (3). Patients with the IPSS-R intermediate risk represent a gray zone including patients with indolent chronic diseases and other who rapidly progress to AML, highlighting the need for further improvement of available tools. Although there is an obvious higher demand for risk assessment in HR-MDS, even LR patients deserve a more accurate prognostic allocation. In fact, they show an intrinsic heterogeneity ranging from mild anemia with nearly normal performance status to life-threatening cytopenias, transfusion dependence, frequent hospitalization, and disability. Therapeutic strategies are still very limited and mainly consist of erythropoiesis stimulating agents (ESAs) and transfusion support for anemic patients (1, 4). Further treatments are indicated only in specific subsets (i.e., lenalidomide for 5q- syndromes, luspatercept in MDS with ring sideroblasts (MDS RS), danazol, thrombopoietin receptor analogues, and immunosuppression in thrombocytopenic and hypoplastic cases) and with responses limited in number and duration (1, 4–7). Finally, the contribution of molecular lesions as well as of autoimmune phenomena to MDS pathogenesis and clinical course, including leukemic evolution, is a field of open investigation (8–15). The objective of this study was to describe the clinical and laboratory features of LR-MDS patients followed at a single tertiary hematologic center. We focused on morphological and molecular aspects, as well as on the presence of markers of autoimmunity, to identify predictors of hematologic improvement and outcome.

## Materials and Methods

### Patients and Hematologic Evaluation

All patients with LR MDS diagnosed from 2001 until the time of writing at a tertiary hematologic center in Milan, Italy were consecutively included in the analysis. The study was conducted according to Helsinki Declaration and was approved by the local ethical committee.

Diagnoses were registered based on the revised 4th edition (2016 update) of the World Health Organization (WHO) Classification of Tumors of Hematopoietic and Lymphoid Tissues (16).

Inclusion criteria encompassed the presence of a low or intermediate-1 IPSS score calculated as per published guidelines (2). Thereafter, patients were re-stratified according to the IPSS-R and kept in the descriptive analyses to further highlight differences in clinical/laboratory features and prognosis (3).

Clinical and laboratory data at diagnosis, including hemolytic markers, levels of endogenous erythropoietin, and creatinine values, were retrospectively collected.

### Immunohematological Data

Immunohematological data were registered, including direct antiglobulin test (DAT) and antiplatelet autoantibodies (anti-PLT antibodies), and correlated with clinical and laboratory findings. In details, DAT for the detection of IgG and complement bound to erythrocytes was performed with the tube technique using the standard method with polyspecific antihuman globulin and monospecific anti-IgG and anti-C3 antisera (Ortho Clinicals Diagnostics). Antibodies antihuman platelet antigen (HPA) were detected by a solid-phase test (Capture-P^®^ Ready Screen, Immucor Inc., Norcross, GA).

Anti-erythroblast autoantibodies were detected in bone marrow cultures as reported elsewhere (17, 18).

Briefly, heparinized blood samples were diluted and either unstimulated or stimulated with phytohemagglutinin, phorbol-12-myristate-13-acetate, and Pokeweed for 48 h. The cultured cell suspension was washed and incubated with peroxidase-conjugated rabbit antihuman IgG (Dako, Glostrup, Denmark) at 37°C for 30 min. About 100 mcL of this mixture was added to the IgG-coated plates and incubated at 37°C for 30 min. The colorimetric reaction was developed by o-phenylenediamine dihydrochloride (Sigma), and the anti-RBC-bound IgG value was calculated referring to a log–log plot curve. The positivity for MS-DAT was defined as a value exceeding the mean plus 3SD of 150 healthy blood donors. In a fraction of patients, the following cytokines were evaluated in serum using commercial ELISA kits (High Sensitivity Elisa kits, Invitrogen by Thermo Fisher Scientific, MA, USA, human TGF-beta ELISA kit, Immunological Sciences, Rome, Italy): interleukin (IL)6, IL10, IL17, tumor necrosis factor (TNF)-alpha, interferon (IFN)-gamma, and transforming growth factor (TGF)-beta. Cytokine levels were compared with 40 age- and sex-matched healthy controls.

Notably, MDS diagnosis predated immunological evaluation and encompassed WHO criteria (16) (including concomitant cytopenia, either Hb<11 g/dL, neutrophils (absolute neutrophil counts, ANC) <1.8x10^9^/L, or platelets (PLT) <100x10^9^/L, and bone marrow dysplasia >10% in at least one lineage, or MDS defining cytogenetics) and the exclusion of secondary causes of cytopenia, particularly chronic inflammatory diseases (including systemic autoimmune conditions), cancer, and nutrient deficiencies. These conditions may show dysplastic features in the bone marrow and a cytopenic phenotype, although not usually reaching the criteria for MDS diagnosis and were not included in the study.

### Bone Marrow Evaluation

For all patients, we collected and recorded bone marrow biopsies reports from diagnosis; cellularity was clearly defined in 159 patients who were included in the analysis. Additionally, for 131 patients, trephine biopsies samples were available at our hospital for re-evaluation by an expert hemopathologist. Histology features were categorized with particular regard to cellularity, dysplastic changes (either numerical, morphologic, and topographic), reticulin fibrosis, and lymphoid infiltrate. Focusing on hypocellularity, we observed that patients with hypoplasia corrected for age as well as those with cellularity <25% independently from age showed the same clinical and laboratory features. Therefore, 25% was used as the cutoff for further analyses, as also reported by Bono et al. (7).

Bone marrow multiparameter flow cytometry (MFC) samples of 136 MDS patients and 25 aplastic anemia cases were retrospectively evaluated by 2 MFC experts. The proportions of the various lymphocyte subpopulations (T, B, and NK cells), monocytes, and mastocytes have been evaluated using the following monoclonal antibodies directed against: CD3, CD10, CD14, CD16, CD19, CD34, CD45, CD56, CD117, and HLA-DR. Finally, classical chromosome banding for cytogenetic analysis was performed in all patients.

### Molecular Analysis

In a subgroup of patients, a NGS study was performed by next-generation sequence technology (Ion Torrents5), Ion Reporters software 5.2, which evaluates the mutational status of 69 potentially oncogenic genes present in the Oncomine Myeloid Research Assay diagnostic panel, specifically hotspot genes (ABL1, BRAF, CBL, CSFR3, DNMT3A, FLT3, GATA2, HRAS, IDH1, IDH2, JAK2, KIT, KRAS, MOL, MYD88, NPM1, NRAS, PTPN11, SETPB1, SF3B1, SRSF2, U2AF1, WT1), full genes (ASXL1, BCOR, CALR, CEBPA, ETV6, EZH2, IKZF1, NF1, PHF6, PRPF8, RB1, RUNX1, SH2B3, STAG2, TET2, TP53, ZRSR2), and fusion transcripts (ABL1, ALK, BCL2, BRAF, CCDN1, CREBBP, EGFR, ETV6, FGFR1, FGFR2, FUS, HMGA2, JAK2, KMT2A, MECOM, MET, MLLT10, MLLT3, MYBL1, MYH11, NTRK3, NUP214, PDGFRA, PDGFRB, RARA, RBM15, RUNX1, TCF3, TFE3). Only variants with an allelic frequency (VAF) > 5% were reported.

In the subgroup of subjects with available molecular status, we applied a new published personalized prediction model to risk-stratify patients with MDS (14), which includes WHO classification, age, Hb levels, platelet, white blood cell, neutrophil, lymphocyte and monocyte counts, bone marrow and peripheral blood blast percentages, cytogenetics according to IPSS-R, number of mutations, and mutational status of the following genes: SF3B1, ASXL1, SRSF2, TP53, STAG2, RUNX1, and RAD21.

### Therapy Evaluation and Outcome Measures

Therapy lines and their efficacy were registered, including recombinant erythropoietin (rEPO), steroids, cyclosporine A (CyA), and danazol. Hematological improvement (HI) to rEPO was calculated according to the revised IWG criteria 2018, as a reduction in red-cell transfusions of ≥4 units per 8 weeks in patients with a baseline transfusion burden of ≥4 units per 8 weeks or as an increase in the hemoglobin level of ≥1.5 g/dL over a period of 8 weeks in patients with a baseline transfusion burden of <4 units per 8 weeks) (19). Overall survival (OS) was calculated from the date of diagnosis until the date of death or the last follow-up. Evolution to acute myeloid leukemia was collected for each patient.

Predictors of HI and OS were assessed, focusing on WHO categories, IPSS and IPSS-R score, hematological values at diagnosis, transfusion dependency, autoimmune positivity, bone marrow cellularity and infiltrate by MFC, and presence and type of mutations by NGS.

### Statistical Analysis

For statistical analysis, Student’s t-test was used for continuous variables and chi-square/Fisher’s exact test for categorical ones. Analysis of variance was performed by using mean, median, ranges, and standard errors. OS was evaluated by the Kaplan–Meier method.

## Results

### Clinical and Hematologic Parameters at Diagnosis

As shown in **Table 1**, 226 patients with LR-MDS followed at our center in the last 20 years were analyzed. Median follow-up was 41.4 months (0.7–226.5). The majority were elderly (83.5% aged > 65 years), with 34 subjects older than 80 years, and there was a slight male predominance (57.5% versus 42.4%). Most patients belonged to three main WHO categories: MDS with single lineage dysplasia (MDS SLD, 31%), with multilineage dysplasia (MDS MLD, 28.3%), and MDS with RS (24.8%). The remaining included myelodysplastic/myeloproliferative neoplasms (MDS/MPN), MDS with isolated 5q-, MDS with excess blasts type 1, and 16 cases initially classified as idiopathic cytopenia/dysplasia of undetermined significance (ICUS/IDUS), but that at the time of referral were re-staged as LR-MDS. Regarding risk and prognostic stratification, almost all patients (77%) were low risk by IPSS and very low (58.8%) or low (34.1%) by IPSS-R. The two patients with MDS with excess of blasts type 1 (MDS EB-1) fell into the intermediate-1 IPSS risk category but were further reclassified as high and very high risk by IPSS-R. Concerning the presenting cytopenia, 96 patients presented with Hb <10 g/dL, 62 with PLT <100 x10^9^/L, and 37 with ANC <1 x10^9^/L (59 had bicytopenia: 29 Hb and PLT, 13 Hb and ANC, and 17 ANC and PLT; and 9 pancytopenia). Median levels of endogenous EPO at diagnosis were 72 U/L (ranging from 1 to 662), below 200 U/L in 139 patients, and >500 U/L in 2. Creatinine values varied greatly from 0.23 to 2.49 mg/dL, and 36 cases showed a decreased glomerular filtration rate (<50 mL/min). Various associations were found among clinical and hematological parameters (**Supplementary Table 1**); in particular, patients with anemia were significantly older (p<0.01), with worse renal function (p=0.03), and had higher endogenous EPO (p=0.001); moreover, subjects with thrombocytopenia more frequently presented lower ANC counts (p=0.01).

**Table 1:** Clinical and laboratory features of low-risk myelodysplastic patients at diagnosis.

	All patients N = 226
**Median age, years (range)**	73.6 (36–90.6)
**Male, N (%)**	130 (57.5)
**Female, N (%)**	96 (42.4)
**MDS type, N (%)**	
MDS-SLD	70 (31)
MDS-MLD	64 (28.3)
MDS with isolated 5q-	8 (3.4)
MDS-RS-SLD	11 (5)
MDS-RS-MLD	45 (20)
MDS/MPN	10 (4.4)
ICUS/IDUS	16 (7)
MDS EB-1	2 (0.9)
**Median Hb, g/dL(range)**	10.4 (5.6–15.5)
**Median ANC x10^9^/L (range)**	2.3 (0.15–13.97)
**Median PLTx10^9^/L (range)**	165 (5–564)
**Median eEPO U/L(range)**	72 (1–662)
**Median LDH, IU/L (range)**	207 (93–703)
**Median creatinine, mg/dL(range)**	0.99 (0.23–2.49)
**IPSS, N (%)**	
Low	174 (77)
int-1	52 (23)
**IPSS-R, N (%)**	
Very low	133 (59)
Low	77 (34.2)
int	14 (6)
High	1 (0.4)
Very high	1 (0.4)
**Cytogenetics, N (%)**	
Normal	176 (78)
Chromosome Y deletions	14 (6.1)
Chromosome 20 deletions	13 (5.6)
Chromosome 5 deletion	11 (5)
Chromosome 11 alteration	4 (1.8)
Trisomy of chromosome 13	2 (0.9)
Trisomy of chromosome 8	4 (1.8)
Translocation (3;4)	1 (0.4)
Complex karyotype	1 (0.4)

IPSS, international prognostic scoring system; IPSS-R, IPSS revised; ANC, absolute neutrophil count; PLT, platelets; eEPO, endogenous erythropoietin; LDH, lactate dehydrogenase; MDS-SLD, myelodysplastic syndrome single lineage dysplasia; MDS-MLD, myelodysplastic syndrome multilineage dysplasia; MDS-RS, myelodysplastic syndrome with ring sideroblast; MDS/MPN, myelodysplastic syndrome/myeloproliferative neoplasm; ICUS/IDUS, Idiopathic cytopenia of undetermined significance and idiopathic dysplasia of uncertain significance; MDS EB-1, myelodysplastic syndrome with excess blasts-1.

Finally, cytogenetics results by classic chromosome banding are summarized in **Table 1** and mainly included deletions of chromosome Y, 20 and 5.

### Morphologic Features

Regarding bone marrow features, the majority of the patients displayed a normo- or hypercellular marrow (89%) while only 11% had a hypocellular trephine. Reticulin fibrosis was present in 11.3% of samples (grade 2 in one patient). As shown in **Table 2**, hypocellular patients showed a slight female predominance and younger age (p=0.001) as compared to normo/hypercellular ones. Moreover, hypocellular patients showed deeper thrombocytopenia [median 114x10^9^/L (30–311) versus 169x10^9^/L (5–564) in normo-hypercellular ones, p=0.002] and neutropenia [1.4x10^9^/L (0.1–4.2) versus 2.3x10^9^/L (0.1–13), p=0.02]. By hemopathologist re-evaluation (**Supplementary Table 2**), the numbers of erythroid and granulocyte precursors and megakaryocytes were significantly reduced in hypocellular patients (p<0.0001). More interestingly, hypocellular cases showed lower frequency of dyserythropoiesis (both topographical and morphological, p=0.003), dysgranulopoiesis (p=0.007), and dysmegakaryopoiesis (p<0.005), and reduced presence of reticulin fibrosis (p=0.01). As regard lymphoid infiltrate, T-cell phenotype was prevalent in all but nine cases who showed equal B- and T-cell infiltrate, and one with higher B-cell population, without remarkable differences according to bone marrow cellularity.

**Table 2:** Clinical and laboratory features of low-risk myelodysplastic patients at diagnosis divided according to bone marrow cellularity.

	Hypocellular (n = 18)	Normo-hypercellular (n = 141)
**Median age, years (range)**	65.9 (48.2–84.8)	75 (56.4–90.6)*
**Male, N (%)**	8 (44.4)	86 (60.9)
**Female, N (%)**	10 (55.6)	55 (39)
**MDS type, N (%)**		
MDS-SLD	2 (11.1)	25 (18)
MDS-MLD	7 (38.8)	53 (38)
MDS with isolated 5q-	3 (16.6)	6 (4.3)
MDS-RS-SLD	1 (5.5)	11 (7.8)
MDS-RS-MLD	0	29 (20.5)
MDS/MPN	0	4 (2.8)
ICUS/IDUS	5 (27.7)	11 (7.8)
MDS EB-1	0	2 (1.4)
**Median Hb, g/dL (range)**	11.5 (8.7–15.5)	10.25 (6.4–14)
**Median ANC x10^9/L (range)**	1.4 (0.37–3.3)	2.19 (0.4–13.9)**
**Median PLTx10^9/L (range)**	118 (30–311)	168 (5–564)***
**Median eEPO U/L (range)**	70 (13.9–220)	48.8 (6–322)
**Median LDH, IU/L (range)**	198 (112–286)	192 (93–427)

*p=0.001, **p=0.02, ***p=0.04.

Only the 159 patients for whom cellularity data were clearly described in bone marrow trephine report were included. ANC, absolute neutrophil count; PLT, platelets; eEPO, endogenous erythropoietin; LDH, lactate dehydrogenase; MDS-SLD, myelodysplastic syndrome single lineage dysplasia; MDS-MLD, myelodysplastic syndrome multilineage dysplasia; MDS-RS, myelodysplastic syndrome with ring sideroblasts; MDS/MPN, myelodysplastic syndrome/myeloproliferative neoplasm; ICUS/IDUS, idiopathic cytopenia of undetermined significance and idiopathic dysplasia of uncertain significance; MDS EB-1, myelodysplastic syndrome with excess blasts-1.

### Cytofluorimetry Analysis of Bone Marrow Microenvironment

**Table 3** shows mean percentages of total lymphocytes and T, B, and NK cells divided according to WHO type: MDS-RS displayed significantly lower B cells and monocytes compared to patients with MDS-SLD (p=0.008 and p=0.03, respectively) and -MLD (p=0.01 for B cells). Elderly patients showed higher mastocyte levels (1.88 ± 1.3% in > 65 years versus 1.2 ± 0.7% in younger patients, p=0.05). Concerning cytopenias, anemic patients (Hb <10 g/dL) showed lower monocytes (3.4 ± 2.2 versus 4.45 ± 3%, p=0.02), thrombocytopenic ones (PLT <100 x10^9/L) showed lower T cells (67.2 ± 15.4 versus 72.4 ± 10.3%, p=0.02) and higher B cells (14.2 ± 11.5 versus 10.5 ± 6.5%, p=0.02), and neutropenic subjects (ANC <1 x10^9/L) displayed higher mastocytes (2.5 ± 2 versus 1.6 ± 1%, p=0.004) and monocytes (5.5 ± 4.08 versus 3.7 ± 2.3%, p=0.004). Hypocellular patients showed increased total lymphocytes (20 ± 9.3 versus 13 ± 7.5%, p<0.0001) and NK cells (21 ± 17 versus 16.6 ± 8%, p=ns), and decreased mastocytes (1.2 ± 1 versus 1.8 ± 1.2%, p=0.03) compared to normo-hypercellular cases. This pattern was more similar to a control group of 25 patients with aplastic anemia who showed higher total lymphocytes and decreased mastocytes as compared to normo-hypercellular MDS patients.

**Table 3:** Lymphoid and myeloid subsets by flow cytometry in low-risk myelodysplastic syndromes (LR-MDS) divided according to WHO classification and in a control group of 25 patients with aplastic anemia.

MDS	Lymphocytes	T cells	B cells	NK cells	Mastocytes	Monocytes
**All patients**	14.2 ± 8	71 ± 12	11.5 ± 8	17.2 ± 10	1.8 ± 1.3	4 ± 2.7
**MDS-SLD**	13.9 ± 6	69.9 ± 10	12.8 ± 6	16.9 ± 10	1.7 ± 1	4.4 ± 2.5
**MDS-MLD**	15.3 ± 9	70.4 ± 13	12.7 ± 8	16.7 ± 9	1.8 ± 1.4	3.8 ± 3
**MDS-RS**	12.4 ± 8	71.9 ± 13	8.8 ± 7	19.1 ± 13	1.7 ± 0.7	3.2 ± 2
**5q-**	17 ± 6	78.6 ± 5	7.7 ± 2	13.8 ± 7	1.5 ± 1	3.8 ± 1.4
**MDS/MPN**	14.3 ± 12	78.2 ± 6	6.2 ± 2	15.6 ± 6	2.3 ± 0.7	9.5 ± 6
**MDS-EB1**	21 ± 1.4	52 ± 28	34.5 ± 39	13.5 ± 11	4.2 ± 5	2.4 ± 1.3
**Aplastic anemia**	22 ± 13.4	71 ± 10.5	12.8 ± 6	16 ± 7.6	0.6 ± 0.5	3 ± 1.3

Values are given as mean ± standard deviation; MDS-SLD, myelodysplastic syndrome single lineage dysplasia; MDS-MLD, myelodysplastic syndrome multilineage dysplasia; MDS-RS, myelodysplastic syndrome with ring sideroblasts; MDS/MPN, myelodysplastic syndrome/myeloproliferative neoplasm; MDS EB1, MDS with excess of blasts-1.

### Autoimmune Features at Baseline

In a subgroup of patients (N=157, 70%), the presence of an autoimmune feature including DAT, anti-PLT, and anti-erythroblast antibodies had been evaluated. Notably, no patients had a previous diagnosis of autoimmune conditions. On the whole, at least one positivity was observed in about half of the patients (46%): DAT positivity in 21.5% (51 tested), anti-PLT in 52% (38 tested), and anti-erythroblasts in 67% (88 tested). Finally, only 1 (3.5%), out of 28 patients tested, showed a PNH clone of 3.5% size on granulocytes. Patients with at least one positivity (**Table 4**) were younger (p=0.001), were predominantly female (p=0.01), were more frequently thrombocytopenic (PLT <100 x10^9/L 38% versus 17%, p=0.02), and more often displayed hypocellular bone marrow (19% versus 9%, p<0.01), with fewer dysplastic changes of granulocytes and megakaryocytes (p<0.01 and p=0.04, respectively) compared to negative cases. Focusing on DAT positive cases (**Supplementary Table 3**), they were younger and with a female prevalence, and mainly belonged to the MDS-SLD or -MLD category (82%). No other distinctive features were noticed, except for a trend for lower PLT, ANC, and bone marrow cellularity. Similar considerations may be drawn for anti-PLT autoantibodies. Focusing on patients with anti-erythroblast autoantibodies, they were predominantly males and mainly belonged to the MDS-SLD or MDS-RS category (49% and 27%, respectively). Moreover, they showed lower Hb levels (median 9.5 g/dL, range 5.6–15 g/dL versus 10.4 g/dL, range 5.6–15.5 g/dL), higher LDH values (225 U/L, range 137–703 U/L versus 207 U/L, range 93–703 U/L), and more frequent hypocellular marrow (18% versus 6%) as compared to negative, cases. Cytokine studies in a subgroup of patients showed that those with anti-erythroblast antibodies displayed reduced T-helper 1 (IFN-γ, TNF-α) and increased T-helper 2/17 profile (IL-6, IL-17, TGF-β), although not significantly (**Table 5**).

**Table 4:** Clinical and laboratory features of low-risk myelodysplastic patients at diagnosis divided according to the positivity of autoimmune tests.

	At least one positive test (N = 37)	All tests negative (N = 57)
**Median age, years (range)**	69 (41.3–89.4)*	76.6 (36–90)
**Male, N (%)**	15 (40.5)	37 (74)
**Female, N (%)**	22 (59.4)**	20 (36)
**MDS type, N (%)**		
MDS-SLD	9 (24.3)	8 (14)
MDS-MLD	15 (40.5)	20 (35)
MDS with isolated 5q-	1 (2.7)	3 (5.2)
MDS-RS-SLD	2 (5.4)	5 (8.7)
MDS-RS-MLD	3 (8.1)	12 (21)
MDS/MPN	0	3 (5.2)
**Laboratory values, median (range)**		
**Hb g/dL**	11 (6.4–14.3)	9.9 (6.6–14.8)
**ANC x10^9^/L**	2.4 (0.37–5.8)	2.2 (0.1–7.2)
**PLTx10^9^/L**	125 (5–370)	156 (20–210)
**Endogenous EPO U/L**	50.5 (6–325)	61.5 (16–566)
**LDH IU/L**	202 (137–703)	198 (112–334)
**Reticulocytes x10^9^/L**	50 (20–68)	50 (20–80)
**Creatinine mg/dL**	0.89 (0.5–1.33)	0.92 (0.23–2.49)
**Bone marrow evaluation**		
**Median cellularity, %**	40 (10–90)	40 (10–90)
Hypocellular, N (%)	7 (18.9)	5 (8.7)
Hypercellular, N (%)	10 (27)	11 (19.2)
Normocellular, N (%)	20 (54)	41 (71.9)
**Reticulin fibrosis, N (%)**	4 (10.8)	9 (15.7)
**Risk scores**		
**IPSS, N (%)**	37	57
Low	26 (70.2)	43 (75.4)
int-1	11 (29.7)	14 (24.5)
**IPSS-R, N (%)**	37	57
Very low	19 (51.3)	31 (54.3)
Low	16 (43.2)	23 (40.3)
int	2 (5.4)	2 (3.5)
High	0	1 (1.7)

At least one positivity refers to the following tests: direct anti-globulin test, anti-platelet antibodies test, and anti-erythroblast antibodies test.

IPSS, international prognostic scoring system; IPSS-R, IPSS revised; ANC, absolute neutrophil count; PLT, platelets; LDH, lactate dehydrogenase; MDS-SLD, myelodysplastic syndrome single lineage dysplasia; MDS-MLD, myelodysplastic syndrome multilineage dysplasia; MDS-RS, myelodysplastic syndrome with ring sideroblasts; MDS/MPN, myelodysplastic syndrome/myeloproliferative neoplasm.

*p=0.001, **p=0.01.

**Table 5:** Bone marrow cytokine levels in low-risk MDS patients with or without anti-erythroblast antibodies.

	Positive	Negative
**Anti-erythroblast antibodies** (ng/mL)	727 ± 136	118 ± 17
**IFN-gamma** (pg/mL)	1,095 ± 146	1,169 ± 54
**TNF-alpha** (pg/mL)	125 ± 33	275 ± 107
**IL-10** (pg/mL)	647 ± 158	886 ± 344
**IL-6** (pg/mL)	71 ± 3	65 ± 4
**IL-17** (pg/mL)	326 ± 122	120 ± 99
**TGF-beta** (pg/mL)	12,666 ± 1,298	10,870 ± 1,939

Mean ± SE of 22 anti-erythroblast antibodies positive and 13 negative patients.

### Molecular Analysis

**Figure 1:**
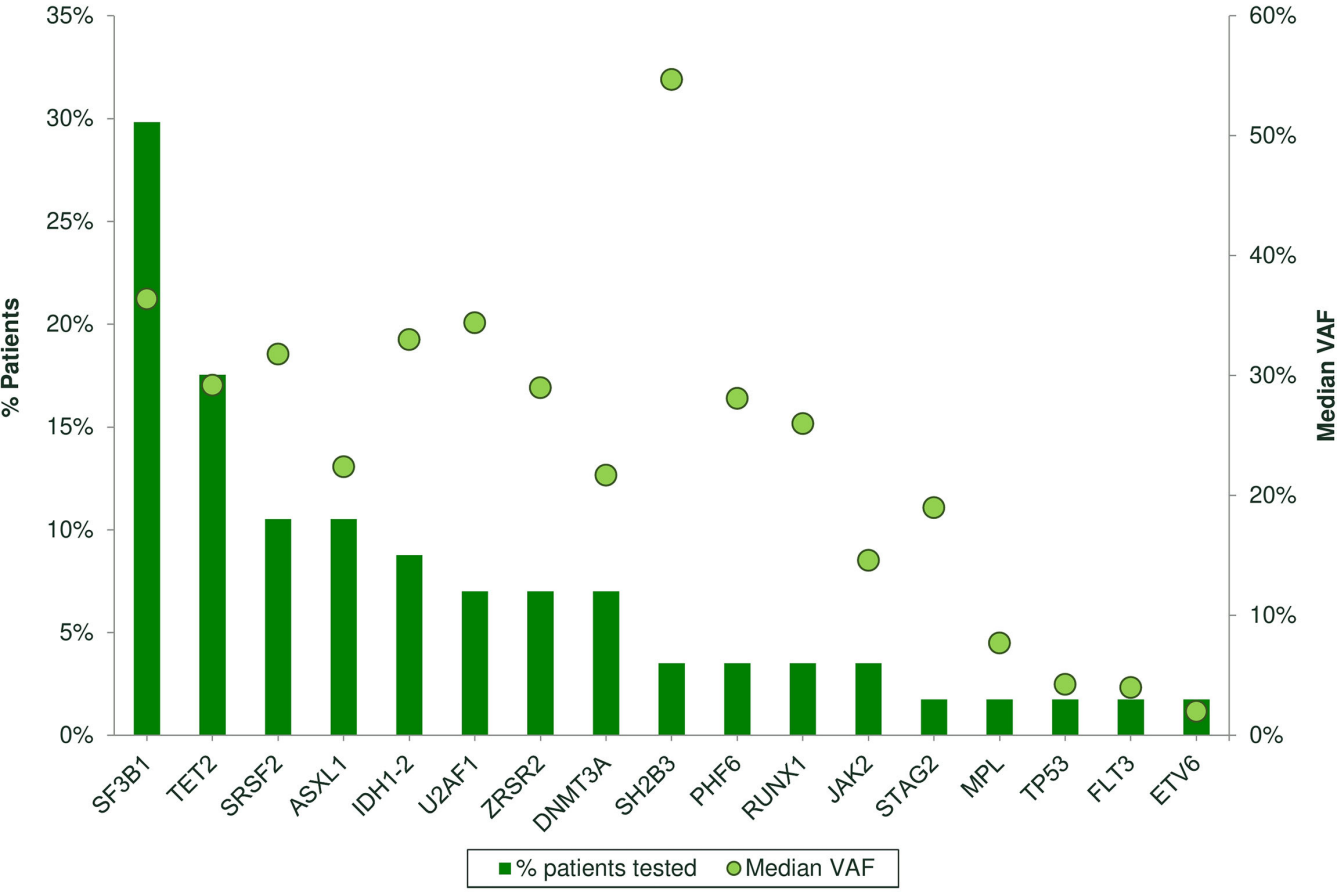
Frequency of somatic mutations by next-generation sequencing (NGS) analysis in 65 patients. Bars represent the % of patients carrying each mutation. Spheres represent the median variant allele frequency (CAF) for each mutation.

**Table 6** shows the clinical features of 65 patients tested for somatic mutations in myeloid genes: 67.7% of the cases showed at least one mutation involving SF3B1 (N=20), TET2 (N=10), DNMT3A (N=4), SRSF2 (N=7), ASXL1 (N=6), ZRSR2 (N=4), IDH2 (N=3), IDH1 (N=2), SH2B3 (N=2), U2AF1 (N=4), P53 (N=1), ETV6 (N=1), PHF6 (N=2), STAG2 (N=1), RUNX1 (N=2), JAK2 V617F (N=2), FLT3 (N=1), and MPL (N=1) with a median VAF of 30% (**Figure 1**). The details of each mutation are reported in **Supplementary Table 4**. Most of the patients presented with splicing mutations (53.8%), followed by DNA methylation (29.2%) and chromatin modifiers (9.2%). Subjects harboring at least one mutation were older as compared to NGS negative cases (p<0.01). Harboring 2 or more mutations (N=22) was associated with older age [76 (41–86) versus 71 (48–85) years, p< 0.05], lower neutrophil counts at diagnosis [1.47 (0.4–4.2) versus 2.71 (0.4–5.8) x10^9^/L, p<0.01], and presence of normo/hypercellular bone marrow (100% versus 82%, p=0.04). The presence of 3 or more mutations (N=11) was associated with deeper neutropenia [ANC 1.1 (0.2–2) versus 2.6 (1.9–5.2) x10^9^/L, p<0.01] and with increased bone marrow lymphocytes by MFC (22.4 ± 14 versus 10.8 ± 3.7%, p<0.001). Specifically, mutated chromatin and transcription factor genes were associated with higher total lymphocytes (25 ± 14 versus 11 ± 6%, p=0.001 and 22.5 ± 16.5 versus 12.3 ± 6.8%, p=0.02, respectively), and mutations of DNA methylation genes were related to lower T cells (69 ± 11 versus 76 ± 9%, p=0.04). Finally, mutations in TP53 or PHF6 were associated with increased T cells (83 + 0.7 versus 73 + 10%, p<0.001), mastocytes (2.4 + 1.4 versus 1.7 + 0.7, p<0.01), and monocytes (7.5 + 5 versus 3.8 + 2.3%, p=0.05), and decreased NK (7 + 1.4 versus 16 + 8, p<0.001). Other associations among laboratory parameters at diagnosis and specific somatic mutations are summarized in **Supplementary Table 5**.

**Table 6:** Clinical and laboratory features of low-risk myelodysplastic patients divided according to the presence of at least one somatic mutation by next-generation sequencing.

	NGS neg (n = 21)	Any mutation (n = 44)
**Median age, years (range)**	71.7 (41.3–82.3)	74.6 (48.2–86)*
**Male, N (%)**	9 (42.8)	27 (61.3)
**Female, N (%)**	12 (57.2)	17 (38.7)
**MDS type, N (%)**		
MDS-SLD	3 (14.3)	7 (15.9)
MDS-MLD	12 (57.1)	13 (29.5)
MDS with isolated 5q-	0 (0)	2 (4.5)
MDS-RS-SLD	2 (9.5)	5 (11.4)
MDS-RS-MLD	1 (4.7)	14 (31.8)
MDS-EB-1	0	1 (2.3)
MDS/MPN	0	2 (4.5)
ICUS/IDUS	3 (14.3)	0
**Laboratory values, median (range)**		
**Hb, g/dL**	10.6 (6.9–14.8)	9.8 (7.6–13.9)
**ANC x10^9^/L**	2.4 (0.9–4.4)	2.1 (0.3–5.8)
**PLTx10^9^/L**	148 (32–320)	162 (25–564)
**Endogenous EPO U/L**	59 (8.2–229)	77.7 (15.5–566)
**LDH, IU/L**	212 (145–313)	191 (118–323)
**Creatinine, mg/dL**	0.9 (0.5–1.68)	0.9 (0.5–1.86)
**Bone marrow evaluation**		
**Median cellularity, %(range)**	40 (20–70)	40 (15–90)
Hypocellular, N (%)	4 (19)	3 (6.8)
Hypercellular, N (%)	5 (23.8)	12 (27.3)
Normocellular, N (%)	12 (57.1)	29 (65.9)
**Reticulin fibrosis, N (%)**	5 (23.8)	3 (6.8)
**Risk scores**		
**IPSS, N (%)**		
Low	18 (85.7)	30 (68.8)
int-1	3 (14.3)	14 (31.2)
**IPSS-R, N (%)**		
Very low	14 (66.6)	22 (50)
Low	7 (33.4)	18 (40.9)
Int	0	3 (6.8)
High	0	1 (2.3)
**Autoimmunity**		
**Autoimmune positivity, N (%)**	5 (23.8)	9 (20.4)
DAT positivity, N (%)	2 (9.5)	5 (11.4)
Anti-PLT positivity, N (%)	3 (14.8)	6 (13.6)
MS-DAT positivity, N (%)	0	1 (2.3)
**Treatment**		
** Treated, N (%)**	8 (38.1)	30 (68.8)
** Transfusions, N (%)**	5 (23.8)	19 (43.2)
**Erythropoietin, N (%)**	8 (38.1)	29 (65.9)
Response, N (%)	5 (62.5)	20 (68.9)
Time to response, months	5.0 (2.3–18)	5.9 (1.4–15.9)

MDS-SLD, myelodysplastic syndrome single lineage dysplasia; MDS-MLD, myelodysplastic syndrome multilineage dysplasia; MDS-RS, myelodysplastic syndrome with ring sideroblasts; MDS/MPN, myelodysplastic syndrome/myeloproliferative neoplasm; ICUS/IDUS, idiopathic cytopenia of undetermined significance and idiopathic dysplasia of uncertain significance; PLT, platelets; ANC, absolute neutrophil count; Hb, hemoglobin; EPO, erythropoietin.

*p<0.01.

### Application of a Personalized Prediction Model Including Molecular Features

The overall survival and probability of leukemic transformation was also evaluated by applying the recently published personalized prediction model. Overall, predicted survival at 24 months was 40.76% (26.6–53.7), lower than that expected per IPSS-R stratification. Additionally, by dividing patients according to IPSS-R categories, the 24-month OS did not differ significantly among very low, low-, and intermediate-risk categories (40%, 32–49.6, vs 41.2%, 28–53.7, vs 43.8%, 42–47.7, respectively, all p >0.3).

### Treatment

Fifty-two patients (32.9%) were red-cell transfusion-dependent at diagnosis (≥6 units/year), without differences according to BM cellularity, positivity of autoimmune markers, or molecular findings. During the follow-up, 79 (49.3%) patients received at least one therapy: all recombinant EPO (71 with epoetin alpha, 6 darbepoetin, median dose 40,000 U/week, range 10,000–80,000 U/week), 15 (14.2%) steroids, 3 lenalidomide for 5q syndrome (3.8%), 3 danazol (3.8%), and 1 CyA (1.2%). The other patients required clinical-laboratory follow-up only due to moderate cytopenia (i.e., Hb ≥10 g/dL, PLT >100x10^9^/L) and absence of disease-specific symptoms. Iron chelation was administered in 16 patients (10%). Sixty patients (75.9%) showed a HI after rEPO with a median time to best response of 4 months (range 1.4–18.2). Expectedly, HI correlated with transfusion independency (92% versus 62%, p=0.002) and with lower baseline endogenous EPO (64 ± 56 versus 109 ± 100 U/L, p=0.01). Additional factors associated with HI were the presence of normo/hypercellular marrow (75% versus 50% in hypocellular cases), of reduced marrow T cells by MFC (69 + 14% versus 76 + 10%, p=0.05), and of increased B cells (13 + 11% versus 7.5 + 5%, p=0.01). Moreover, patients with at least one positivity of autoimmune tests and those with positive DAT more frequently responded to rEPO (57% for any positivity and 50% for DAT versus 20% for negative cases), as did cases with normal karyotype (83% versus 56%, p=0.01). Regarding NGS analysis, patients with at least 2 mutations showed lower response to rEPO (52 versus 79%), although not significantly. Concerning patients treated with steroids, most (66.6%) were transfusion-dependent at diagnosis, 87% had been treated with rEPO as first line (47% responding), and 33.3% obtained a HI. Finally, one patient was treated with CyA for thrombocytopenic hypoplastic MDS and responded, 3 subjects received danazol due to either anemia (N=1) or thrombocytopenia (N=2) and only 1 responded, and 1 patient received eltrombopag with no response.

### Leukemic Evolution and Survival

**Figure 2:**
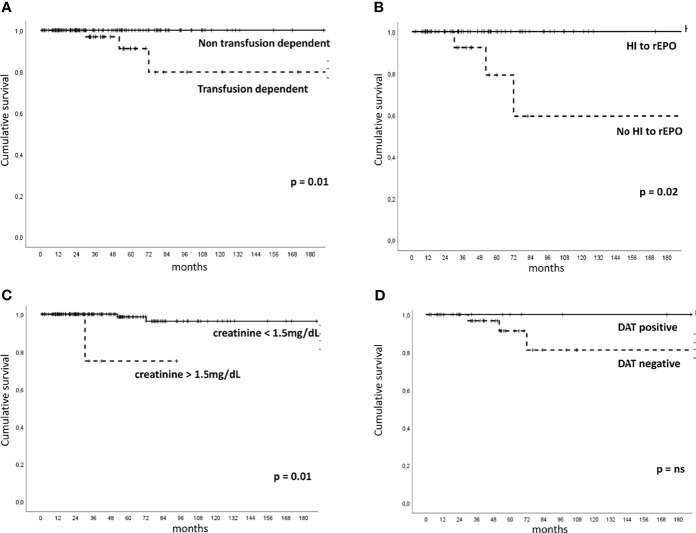
Overall survival in patients with low-risk myelodysplastic syndrome according to clinical-laboratory features. Kaplan–Meier for to transfusion dependency [**(A)**, number of patients at risk 159], hematologic improvement (HI) to recombinant erythropoietin (rEPO) [**(B)**, number of patients at risk 79], creatinine values [**(C)**, number of patients at risk 159], and direct antiglobulin test (DAT) positivity [**(D)**, number of patients at risk 51]. ns, not significant.

Five patients (2.2%) evolved to acute myeloid leukemia after a median follow-up of 72 months (51–214). They were mainly females, transfusion-dependent, unresponsive to rEPO (one had also received steroids, danazol, and eltrombopag), and with normo/hypercellular bone marrow (one of them showing MDS/MPN overlap morphology). Moreover, 3 of them presented anti-PLT autoantibodies, 1 positive DAT, and 4 displayed ≥2 somatic mutations, involving ASXL1, ETV6, and RUNX1. Five (3.1%) patients died, three for AML evolution and two for pneumonia and life-threatening cytopenias. By Kaplan–Meier analysis (**Figure 2**), median OS positively correlated with achievement of HI to rEPO (216 months, 95% CI 211–221 versus 154 months, 92–216, p=0.02) and with transfusion independence (217 months, 213–221 versus 187 months, 150–224, p=0.01). Contrarily, patients with creatinine values >1.5 mg/dL showed shorter OS (77 months, 50–103 versus 214 months, 204–224, p=0.01). Finally, DAT positivity well separated survival curves (217 months, 213–221 versus 194 months, 160–229 for DAT negative cases), although OS diversity was not statistically significant.

## Discussion

**Figure 3:**
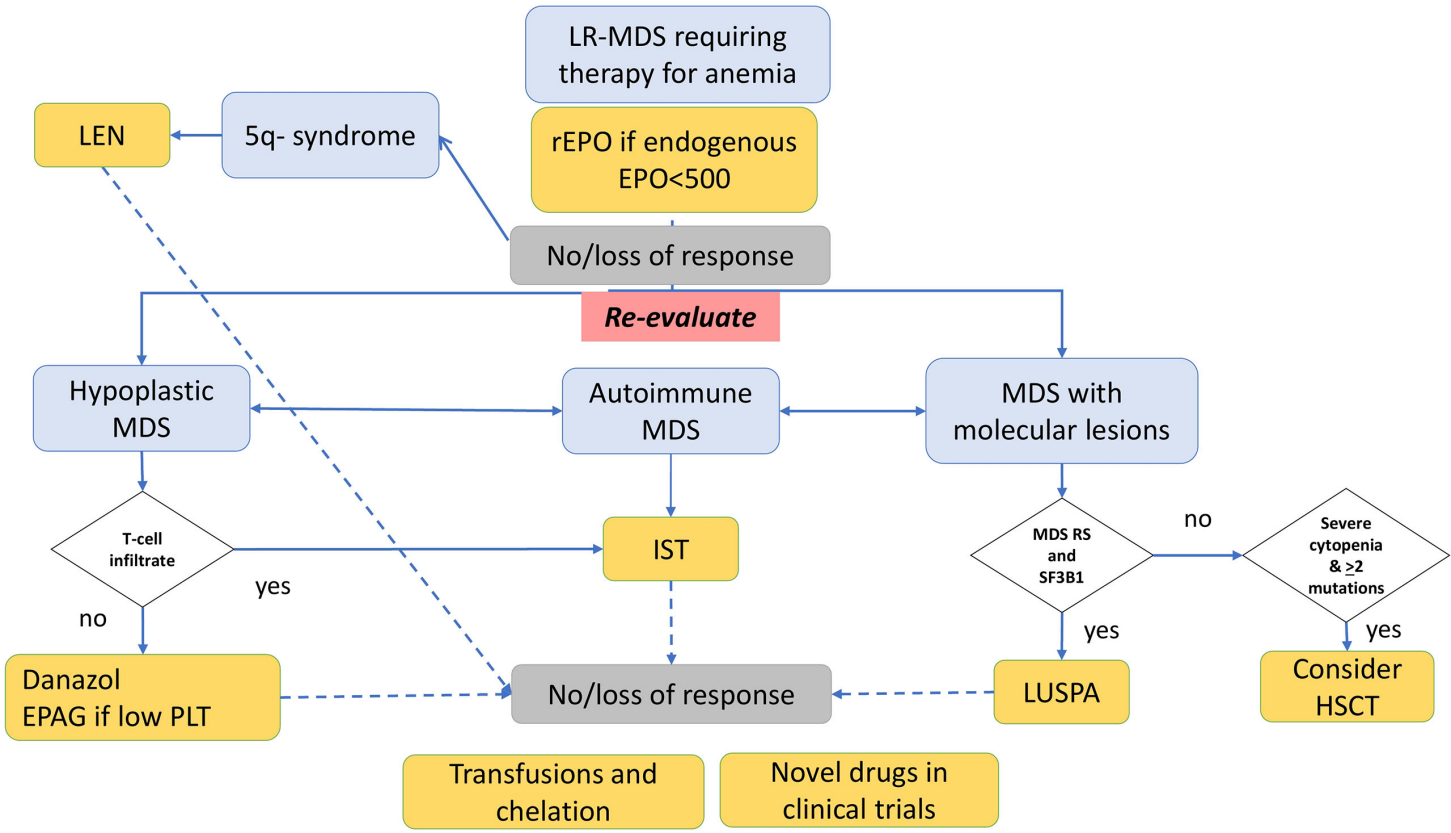
Management of low-risk myelodysplastic syndrome (LR-MDS) failing recombinant erythropoietin (rEPO) according to morphologic, autoimmune, and molecular features. Patients failing rEPO should undergo re-evaluation. Those with 5q- syndrome may benefit from lenalidomide; those with hypoplastic MDS and T-cell bone marrow infiltrate may be candidates to immunosuppression (IST) with cyclosporine and steroids; those without T-cell infiltrate may be treated with danazol or, if thrombocytopenic, with eltrombopag in the clinical trial. Patients with positive anti-erythrocyte, antiplatelet, or anti-erythroblast antibodies may benefit from IST with steroids. Those with SF3B1 mutation may respond to luspatercept (LUSPA), and those with life-threatening cytopenias and ≥2 mutations should be assessed for HSCT if feasible. Nonresponders are candidate for transfusion support with iron chelation and enrollment in clinical trials with novel drugs.

Here we describe a single center series of LR-MDS patients and confirm a great clinical heterogeneity, regarding the prevailing cytopenia, morphologic and immunologic features, therapy requirement, and outcome. Despite the efforts made to redraw risk scores for MDS patients, including the IPSS-R and the WHO classification‐based Prognostic Scoring System (WPSS) (3–22), in a recent analysis of 1,290 individuals, none of these indices was able to identify patients with poor prognosis among low IPSS scores (23). This is particularly frustrating given the difficulty to allocate LR patients to transplant and the advent of novel drugs, which advocates for better stratification and predictors of response. In our experience, we dissected granular clinical and hematologic features of LR-MDS, but the final picture is still puzzled, and patients ended up being managed on a case-by-case basis. In fact, while the diagnostic, therapeutic, and prognostic role of classic cytogenetics is undoubtful, other factors are still object of debate (1, 7, 24). We propose that the evaluation of pathophysiologic aspects including bone marrow hypoplasia, markers of autoimmune activation, and molecular lesions may provide hints for treatment allocation after rEPO failure (**Figure 3**). Regarding the first, bone marrow cellularity allows the identification of hypoplastic MDS (younger, with lower morphologic and topographic dysplasia, and higher bone marrow lymphoid infiltrate) (7, 25, 26) who will potentially benefit from immunosuppressive treatment with anti-thymocyte globulin (ATG) and cyclosporine in up to 30% of cases (26–29). Concerning autoimmunity features, the frequency of autoantibodies (DAT, anti-PLT autoantibodies, or anti-erythroblast antibodies) in the present study was substantial, also in keeping with the experience of our center in managing autoimmune cytopenias. This is, however, in line with several reports describing autoimmunity markers in more than 30% of MDS subjects, particularly if systematically tested. The latter were associated with a more cytopenic phenotype in ours and other experiences (30–34). Although overt autoimmune manifestations are reported in 4–8% of MDS only, immunosuppression with steroids might be useful in LR-MDS with markers of autoimmunity (“autoimmune” MDS), particularly in thrombocytopenic patients, but even in anemic ones if hemolytic markers are altered. Accordingly, therapy with thrombopoietin receptor agonists (TPO-RA), labeled for immune thrombocytopenia and aplastic anemia, has shown some efficacy in thrombocytopenic MDS patients (6). Moreover, it has been shown that half of the cases of LR-MDS display anti-erythroblast antibodies, and that bone marrow culture supernatants of positive cases were able to induce dysplastic changes in normal BM (17, 18). Additionally, patients with anti-erythroblast antibodies displayed reduced T-helper 1 cytokines (IFN-γ, TNF-α) and increased T-helper 2/17 profile (IL-6, IL-17, TGF-β), consistent with prominent humoral immunity and reduced pro-inflammatory microenvironment. This may result in a more preserved marrow stemness, which was associated with a better response to rEPO, similarly to that observed in AIHA (35). In this context, a deeper knowledge of the immunological microenvironment (lymphoid, monocyte, mastocyte infiltrate, etc.), along with the cytokine profile, may contribute to identify candidates to novel therapies targeting cytokine patterns and immunological checkpoints in the future (36, 37). Another immunological signature is PNH positivity, resulting from the acquisition of PIG-A mutation and autoimmune stem cell attack. In our series, one patient harbored a small PNH clone, resulting in a lower prevalence than previously reported (38–40), likely due to the threshold of 1% clone size used at our laboratory. She responded well to CyA, in agreement with several reports that correlated small and very small clones with better response to IST (38–40). Although the clinical significance of small and very small clones in bone marrow failures and MDS is an object of debate, PNH testing at diagnosis in this setting, particularly in case of increased LDH values, is largely agreed upon (39, 40).

Regarding molecular features, they are being actively investigated to improve prognostication (14, 15). However, cytogenetics and blast counts are still the mainstay, and mutational status showed controversial impact, particularly in low-risk MDS patients apart from SF3B1 mutation (1, 8–15, 41). In our study, the application of a recently published model including the number of mutations and the mutational status of 7 genes (14) did not further stratified patients and predicted survival probability did not match that of our cohort (5 deaths over a 72-month follow-up period). In any case, the mutational burden was related to older age, the presence of a more cytopenic phenotype (particularly neutropenic) with higher bone marrow cellularity, and lymphoid infiltrate. The latter was particularly high in patients harboring mutations of DNA methylation and chromatin modifier genes and was also related to a lower response to r-EPO. These findings suggest that myeloid cells with highly disrupted genetic machinery fail to differentiate under growth factor stimulation. Additionally, all patients who evolved to AML had 2 or more somatic mutations, possibly suggesting a transplant strategy, if feasible, in the presence of refractory life-threatening cytopenia. Finally, patients evolving to AML presented a marker of autoimmunity, but DAT positivity was associated with a trend for better survival. This paradox may be speculatively related to the transition of the immune system from pro-inflammatory/pro-apoptotic in early MDS to tumor permissive facilitating leukemic progression in the late stage (26, 42). Indeed, somatic mutations may be present not only in the myeloid compartment but also in the immunologic microenvironment, as described for MDS, but also for aplastic anemia and other autoimmune diseases (43–45). Lymphoid clonality, particularly of CD8+ T-cells and large granular lymphocytes (LGL) in patients with bone marrow failure, may represent both a player in inducing apoptosis of marrow precursors and a result of chronic stimulation *via* exposure of self-antigens resulting from ineffective erythropoiesis. In this view, it has been reported that STAT3-mutant LGL clones may facilitate bone marrow failure in a subset of aplastic anemia patients and may be potentially amenable to immunosuppressive treatment (44, 45).

Although this study carries several limitations, particularly regarding the retrospective nature of the analysis, it provides the snapshot of a *real-world* series of patients with LR MDS managed at the state of the art for 2022, including the application of novel molecular risk scores; it highlights the unmet needs of this patient population and underlines the importance of integrating immunological, bone marrow, and molecular features to improve patient care.

## Conclusions

In conclusion, our results reflect the complex interplay among bone marrow stemness, dysplasia/genetic lesions, and the immunologic microenvironment in LR-MDS. Particularly, the presence of autoantibodies may identify a subgroup of MDS patients who, along with hypoplastic cases, may benefit from an immunosuppressive approach. Still, transfusion dependence and refractoriness to r-EPO regroup patients with patients with particularly dismal outcome representing a true unmet need. Several pathogenic actors in MDS may be targeted by different therapeutic agents, including IST, TPO-RA, and new biologic drugs. Only considering this biologic diversity, the clinical management of LR-MDS patients may be optimized in the near future.

## Data Availability Statement

The original contributions presented in the study are included in the article/**Supplementary Material**. Further inquiries can be directed to the corresponding author.

## Ethics Statement

The study was conducted according to Helsinki Declaration, was approved by the local Ethical Committee, and patients gave informed consent.

## Author Contributions

BF, GL, JG, and WB designed the study, followed the patients, collected the data, and wrote the paper. GCa collected molecular data and wrote the paper. LC and MB performed the cytofluorimetry on bone marrow samples. AZ tested anti-erythroblasts and performed cytokine studies. GCr performed the pathologic revision of bone marrow trephines. NR performed the immunohematological tests. All authors revised the manuscript for important intellectual content and approved the final version.

## Conflict of Interest

BF received consultancy from Apellis, Momenta, and Novartis and lecture fee/congress support from Alexion and Apellis. WB received consultancy from Agios, Alexion, Apellis, Biocryst, Bioverativ, Incyte, Momenta, and Novartis; and lecture fee/congress support from Alexion, Incyte, Novartis, and Sanofi.

The remaining authors declare that the research was conducted in the absence of any commercial or financial relationships that could be construed as a potential conflict of interest.

## Publisher’s Note

All claims expressed in this article are solely those of the authors and do not necessarily represent those of their affiliated organizations, or those of the publisher, the editors and the reviewers. Any product that may be evaluated in this article, or claim that may be made by its manufacturer, is not guaranteed or endorsed by the publisher.
