# Supplementary material for: Low-Risk Myelodysplastic Syndrome Revisited: Morphological, Autoimmune, and Molecular Features as Predictors of Outcome in a Single Center Experience
Source: Front Oncol. 2022 Mar 22;12:795955. doi: 10.3389/fonc.2022.795955 (PMC8980524; doi:10.3389/fonc.2022.795955)
Supplement: Supplementary file 1 [file DataSheet_1.docx]

| **Age** | **Age at diagnosis < 65y** | **Age at diagnosis >65y** | **p** |
| --- | --- | --- | --- |
| **Mean Hb +DS(g/dL)** | 11,5 +2.2 | 10,4+1.8 | 0,01 |
| **Mean ANC +DS x10^9/L** | 2.5+0.9 | 1.8+1.7 | 0,03 |
| **Mean creatinine +DS (mg/dL)** | 0,8+0.2 | 1+0.3 | 0,02 |
| **Anemia** | **Hb at diagnosis < 10 g/dL** | **Hb at diagnosis > 10 g/dL** | **p** |
| **Mean age at diagnosis +DS (years)** | 76+8 | 71,4+9 | 0,01 |
| **Mean creatinine +DS (mg/dL)** | 1,02+ 0.3 | 0,9+0.4 | 0,03 |
| **Mean ANC +DS x10^9/L** | 2.7+1.4 | 2.2+1.8 | 0,05 |
| **Mean endogenous EPO +DS (U/L)** | 104+121 | 49+45 | 0,001 |
| **Thrombocytopenia** | **PLT at diagnosis <100x10^9/L** | **PLT at diagnosis > 100x10^9/L** | **p** |
| **Mean Hb +DS (g/dL)** | 11.2+2.1 | 10,3+1.8 | 0,09 |
| **Mean ANC +DS x10^9/L** | 2+1 | 2.5+1.8 | 0,01 |
| **Mean LDH +DS (u/L)** | 200+70 | 228+67 | 0,02 |

**Supplementary table 1: clinical and laboratory parameters showing significant associations.**

Hb hemoglobin; ANC absolute neutrophil count; PLT platelets; EPO endogenous erythropoietin; LDH lactate dehydrogenase; DS standard deviation

**Supplementary table 2: bone marrow histology revision in a subgroup of 130 patients, altogether and divided according to bone marrow cellularity.**

|  | **All patients (n=130)** | **Hypocellular (n=19)** | **Normo-hypercellular (n=111)** |
| --- | --- | --- | --- |
|  |  |  |  |
| **Median cellularity % range** | 40 (10-90) | 20 (10-20) | 45 (30-90) |
| **Cellularity/age N(%)** |  |  |  |
| Reduced | 19(15) | 19 (100) | 0 |
| Normal | 62 (48) | 0 | 62 (56) |
| Augmented | 49 (37) | 0 | 49 (44) |
| **Disomogenous cellularity N(%)** | 57 (44) | 13 (68) | 44 (40) |
| **Reticulin fibrosis N(%)** | 24 (18) | 0 | 24 (22)** |
| **Erythroid series N(%)** |  |  |  |
| Reduced | 9 (7) | 9 (47)* | 0 |
| Normal | 64 (49) | 10 (53) | 54 (49) |
| Augmented | 57 (44) | 0 | 57 (51) |
| **Topological Dyserythropoiesis N(%)** | 109 (84) | 11 (58) | 98 (88)* |
| **Morphological dyserythropoiesis N(%)** | 97 (75) | 11 (58) | 86 (77)* |
| **Granulocytes quantity N(%)** |  |  |  |
| Reduced | 36 (28) | 17 (89)* | 19 (17) |
| Normal | 72 (55) | 2 (11) | 70 (63) |
| Augmented | 22 (17) | 0 | 22 (20) |
| **Granulocytes maturation N(%)** |  |  |  |
| Normal | 40 (31) | 11 (58) | 29 (27)* |
| lrregular | 73 (56) | 7 (37) | 66 (59) |
| Left shift | 17 (13) | 1 (5) | 16 (14) |
| **Megakaryocytes features N(%)** |  |  |  |
| Reduced | 6 (5) | 6 (32) | 0 |
| Normal | 60 (46) | 12 (63) | 48 (43) |
| Augmented | 64 (49) | 1 (5)* | 63 (57) |
| **Megakaryocytes type N(%)** |  |  |  |
| clusters | 50 (38) | 1 (5) | 49 (44)* |
| dystrophic | 37 (28) | 1 (5)* | 36 (32) |
| hypolobated | 4 (3) | 0 | 4 (4) |
| small | 32 (25) | 13 (68)* | 19 (17) |
| small and hypolobated | 16 (12) | 1 (5) | 15 (14) |
| matures | 30 (23) | 3 (17) | 27 (24) |
| MPN-lyke | 10 (8) | 0 | 10 (9) |
| multinucleated | 1(0.7) | 1(5) | 0 |
| **Lymphoid pattern N(%)** |  |  |  |
| interstitial | 101 (78) | 17 (90) | 84 (76) |
| nodular interstitial | 29 (22) | 2 (10) | 27 (24) |
| **Lymphoid inflitrate N(%)** |  |  |  |
| <5% | 89 (99) | 12 (63) | 77 (69) |
| >5% | 41 (31) | 7 (37) | 34 (31) |
| *p<0.01, **p=0.01 |  |  |  |

**Supplementary table 3: clinical and hematologic features according to positivity of the direct antiglobulin test (DAT) and anti-platelets antibodies (anti-PLT) positivity.**

|  | **DAT+ (N=11)** | **DAT- (N=40)** | **anti-PLT+ (N=20)** | **anti-PLT-(N=18)** |
| --- | --- | --- | --- | --- |
| **Median age, years (range)** | 69.5 (41.3-82.4) | 75.2 (36-90) | 68.4 (48.2-89.4) | 75.4 (60.4-80.3) |
| **Male, N(%)** | 4 (36.3) | 25 (62.5) | 8 (40) | 11 (61) |
| **Female, N(%)** | 7 (63.6) | 15 (37.5) | 12 (60) | 7 (38.8) |
| **MDS type, N(%)** |  |  |  |  |
| MDS-SLD | 2 (18) | 3 (7.5) | 7 (35) | 3 (16.6) |
| MDS-MLD | 7 (63.6) | 12 (30) | 8 (40) | 11 (61) |
| MDS with isolated 5q- | 1 (9) | 2 (5) | 1 (5) | 0 |
| MDS-RS-SLD | 1 (9) | 4 (10) | 0 | 1 (5) |
| MDS-RS-MLD | 0 | 14 (35) | 1 (5) | 0 |
| MDS/MPN | 0 | 2 (5) | 0 | 1 (5) |
| **Laboratory values, median (range)** | | | | |
| **Hb g/dL** | 11 (6.4-13.9) | 9.45 (7.2-14.3) | 12.5 (7.8-14.3) | 12.7 (6.6-14.8) |
| **ANC x10^9/L** | 1.7(0.4-3.5) | 2.4(0.155.2) | 1.9 (0.37-3.2) | 2.1 (0.2-4.4) |
| **PLTx10^9/L** | 166 (25-370) | 185(32-448) | 91 (5-230) | 66 (28-178) |
| **Endogenous EPO U/L** | 98.6 (8-322) | 57(17-566) | 42 (6-325) | 59 (16-134) |
| **LDH UI/L** | 178 (141-427) | 198(139-334) | 204 (138-393) | 209 (112-287) |
| **Reticulocytes x10^9/L** | 52 (20-200) | 50 (10-60) | 48 (21-210) | 47 (39-199) |
| **creatinine mg/dL** | 0.95 (0.5-1.2) | 0.92(0.58-2.49) | 0.8 (0.5-1.23) | 0.99 (0.5-2.38) |
| **Bone marrow evaluation** | | | | |
| **Median cellularity, %** | 40 (20-90) | 43 (20-90) | 30 (15-70) | 30 (10-70) |
| hypocellular, N(%) | 1 (9) | 2 (5) | 5 (25) | 3 (16,6) |
| hypercellular, N(%) | 2 (18) | 11 (27,5) | 3 (15) | 2 (11,1) |
| normocellular, N(%) | 8 (72,7) | 27 (67,5) | 12 (60) | 13 (72,2) |
| **Reticulin fibrosis, N(%)** | 1 (9) | 7 (17,5) | 1 (5) | 3 (16,6) |
| **Risk scores** | | | | |
| **IPSS, N(%)** | 11 | 40 | 20 | 18 |
| Low | 8 (72,7) | 31 (77,5) | 13 (65) | 12 (66,6) |
| int-1 | 3 (27,2) | 9 (22,5) | 7 (35) | 6 (33,3) |
| **IPSS-R, N(%)** | 11 | 40 | 20 | 18 |
| very low | 4 (36,3) | 24 (60) | 10 (50) | 8 (44,4) |
| low | 6 (54,5) | 15 (37,5) | 9 (45) | 9 (50) |
| int | 1 (9) | 1 (2,5) | 1 (5) | 1 (5,5) |
| MDS SLD myelodysplastic syndrome single lineage dysplasia; MDS MLD myelodysplastic syndrome multilineage dysplasia; MDS-RS myelodysplastic syndrome with ring sideroblasts; MDS/MPN myelodysplastic syndrome/myeloproliferative neoplasm | | | | |

**Supplementary table 4. Details of somatic mutations identified at next generation sequencing (NGS) analysis in 44 patients with myelodysplastic syndrome.**

| **Patient #** | **Diagnosis** | **CHR** | **Type** | **Gene** | **c.** | **p.** | **Annotation** |
| --- | --- | --- | --- | --- | --- | --- | --- |
| 1 | MDS-RS-MLD | 2 | Substitution | SF3B1 | c.2098A>G | p.Lys700Glu | oncogenic |
| 2 | MDS-MLD | 2 | Substitution | SF3B1 | c.2098A>G | p.Lys700Glu | oncogenic |
| 2 | MDS-MLD | 2 | Substitution | DNMT3A | c.1677C>A | p.Cys559Ter |  |
| 3 | MDS-SLD | 4 | Deletion | TET2 | c.368delG | p.(Arg123LeufsTer5) |  |
| 3 | MDS-SLD | 17 | Substitution | SRSF2 | c.284C>A | p.(pro95His) | oncogenic |
| 4 | MDS-RS-MLD | 12 | Unknown | ETV6 | unknown | unknown |  |
| 4 | MDS-RS-MLD | 20 | Unknown | ASXL1 | unknown | unknown |  |
| 4 | MDS-RS-MLD | 21 | Unknown | U2AF1 | unknown | unknown |  |
| 5 | MDS-RS-MLD | 2 | Substitution | SF3B1 | C.1866G>C | p.(Glu622Asp) | oncogenic |
| 6 | MDS-RS-MLD | 2 | Substitution | SF3B1 | c.1868A>G | p.(Tyr623Cys) |  |
| 7 | MDS-RS-SLD | 2 | Substitution | SF3B1 | c.2098A>G |  | oncogenic |
| 8 | MDS-MLD | 4 | Unknown | TET2 | unknown | unknown |  |
| 8 | MDS-MLD | 15 | Unknown | IDH2 | unknown | unknown |  |
| 8 | MDS-MLD | 17 | Unknown | SRSF2 | unknown | unknown |  |
| 9 | MDS-MLD | 2 | Substitution | IDH1 | c.395G>A |  | oncogenic |
| 9 | MDS-MLD | 20 | Substitution | ASXL1 | c.2893C>T |  |  |
| 9 | MDS-MLD | 21 | Substitution | U2AF1 | c.470A>C |  | oncogenic |
| 10 | MDS-MLD | 11 | Substitution | CBL | c.1259G>A | p.(Arg420Gln) | oncogenic |
| 11 | MDS-RS-MLD | 2 | Substitution | SF3B1 | c.2098A>G |  | oncogenic |
| 12 | MDS-RS-MLD | 2 | Substitution | SF3B1 | c.2098A>G |  | oncogenic |
| 12 | MDS-RS-MLD | 4 | Deletion | TET2 | c.3393_3996delTCTT |  |  |
| 12 | MDS-RS-MLD | 20 | Deletion | ASXL1 | c.4322delA |  |  |
| 13 | MDS-MLD | X | Deletion | ZRSR2 | c.918delG | p.(Trp307Gly*) |  |
| 14 | MDS-RS-MLD | 2 | Substitution | SF3B1 | c.2098A>G | p.Lys700Glu | oncogenic |
| 15 | MDS-SLD | X | Deletion | PHF6 | c.94delC | p.(Leu32*) |  |
| 16 | MDS-MLD | 17 | Substitution | SRSF2 | c.284C>T | p.Pro95Leu | oncogenic |
| 17 | MDS-RS-MLD | 2 | Substitution | SF3B1 | c.2098A>G |  | oncogenic |
| 17 | MDS-RS-MLD | 4 | Substitution | TET2 | c.4546C>T |  | oncogenic |
| 18 | MDS-SLD | 2 | Deletion | DNMT3A | c.2666_2682del | p.(Leu899ArgfsTer26) | oncogenic |
| 19 | MDS-MLD | 4 | Insertion | TET2 | c.3139_3140insA | p.Thr104AsnfsTer11 |  |
| 19 | MDS-MLD | X | Deletion | ZRSR2 | c.22delA | p.Thr8ArgfsTer15 |  |
| 20 | MDS-MLD | 2 | Substitution | IDH1 | c.395G>A | p.Arg132His | oncogenic |
| 20 | MDS-MLD | 20 | Deletion | ASXL1 | c.1900_1922 |  |  |
| 20 | MDS-MLD | 21 | Substitution | U2AF1 | c.470A>C | p.Glu157Pro | oncogenic |
| 21 | MDS-MLD | 21 | Substitution | RUNX1 | c.1047T>A | p.(Tyr349*) |  |
| 21 | MDS-MLD | 17 | Substitution | SRSF2 | c.284C>G | p.(Pro95Arg) | oncogenic |
| 21 | MDS-MLD | 20 | Substitution | ASXL1 | c.2278C>T | p.(Gln760*) | oncogenic |
| 22 | MDS-EB1 | 21 | Substitution | RUNX1 | c.484A>G |  |  |
| 22 | MDS-EB1 | X | Substitution | STAG2 | c.646C>T |  |  |
| 22 | MDS-EB1 | X | Insertion | STAG2 | c.872_872 insG |  |  |
| 23 | MDS-SLD | 4 | Deletion | TET2 | c.1263delA | p.(Gly422Glufs*5) | oncogenic |
| 24 | MDS-MLD | 4 | Substitution | TET2 | c.1648C>7 |  | oncogenic |
| 24 | MDS-MLD | 4 | Substitution | TET2 | c.4393C>T |  |  |
| 24 | MDS-MLD | 17 | Substitution | TP53 | c.818G>A |  | oncogenic |
| 25 | MDS-RS-MLD | 2 | Substitution | SF3B1 | c.1873C>7 |  | oncogenic |
| 25 | MDS-RS-MLD | 12 | Substitution | SH2B3 | c.962C>G |  |  |
| 26 | MDS/MPN-RS-T | 2 | Substitution | SF3B1 | c.1997A>G | p.Lys666Arg | oncogenic |
| 26 | MDS/MPN-RS-T | 9 | Substitution | JAK2 | c.1849G>T | p.Val617Phe | oncogenic |
| 27 | MDS-MLD | 21 | Substitution | U2AF1 | c.101C>T | p.Ser34Phe |  |
| 28 | MDS-RS-SLD | 4 | Deletion | TET2 | c.1337delT | p.(Leu446*) |  |
| 28 | MDS-RS-SLD | 17 | Substitution | SRSF2 | c.284C>T | p.(Pro95Leu) | oncogenic |
| 29 | MDS-SLD | 2 | Substitution | SF3B1 | c.1866 G>T |  | oncogenic |
| 30 | MDS-MLD | 4 | Substitution | TET2 | c.1612C>T |  |  |
| 30 | MDS-MLD | 4 | Substitution | TET2 | c.5618T>C |  | oncogenic |
| 30 | MDS-MLD | 17 | Substitution | SRSF2 | c.284C>A |  | oncogenic |
| 31 | MDS-SLD | 2 | Substitution | DNMT3A | c.1843C>T | p.(GIn615) |  |
| 32 | MDS-RS-MLD | 2 | Substitution | SF3B1 | c.2098A>G |  | oncogenic |
| 33 | MDS-SLD | X | Substitution | ZRSR2 | c.1119C>G | p.(Tyr373*) |  |
| 34 | MDS with 5q- | 2 | Unknown | DNMT3A | unknown | unknown |  |
| 34 | MDS with 5q- | 9 | Substitution | JAK2 | c.1849G>T | p.Val617Phe | oncogenic |
| 35 | MDS-RS-MLD | 2 | Substitution | DNMT3A | c.2207G>T | p.(Arg376Leu) | oncogenic |
| 35 | MDS-RS-MLD | 2 | Substitution | SF3B1 | c.1998A>G | p.(Lys666Arg) | oncogenic |
| 35 | MDS-RS-MLD | 4 | Substitution | TET2 | c.44011T>A | p.(Tyr1337Ter) |  |
| 36 | MDS-MLD | 2 | Substitution | SF3B1 | c.2098A>G | p.K700E | oncogenic |
| 36 | MDS-MLD | 1 | Substitution | MPL | c.1514G>A | p.S505N | oncogenic |
| 37 | MDS-MLD | 15 | Substitution | IDH2 | c.419G>A | p.(Arg140Gln) | oncogenic |
| 37 | MDS-MLD | 17 | Substitution | SRSF2 | c.284C>T | p.(Pro95Leu) | oncogenic |
| 38 | MDS-SLD | 13 | Substitution | FLT3 | c.2504A>T | p.Asp835Val, D835V |  |
| 38 | MDS-SLD | 4 | Substitution | TET2 | c.1669C>T | p.GIn557Ter |  |
| 38 | MDS-SLD | 4 | Deletion | TET2 | c.3279_3281 delCCAA | p.Thr1093LysfsTer12 |  |
| 38 | MDS-SLD | 4 | Substitution | TET2 | c.5104C>T | p.GIn1702Ter |  |
| 38 | MDS-SLD | X | Substitution | ZRSR2 | c.868C>T | p.Arg290Ter |  |
| 38 | MDS-SLD | X | Deletion | PHF6 | c.70delA | p.Arg24GlufsTer9 |  |
| 39 | MDS-RS-MLD | 2 | Substitution | SF3B1 | c.1874G>T | p.Arg625Leu | oncogenic |
| 40 | MDS-RS-MLD | 2 | Substitution | SF3B1 | c.2098A>G |  | oncogenic |
| 40 | MDS-RS-MLD | 4 | Insertion | TET2 | c.3397_3398ons |  |  |
| 40 | MDS-RS-MLD | 15 | Substitution | IDH2 | c.419G>A |  | oncogenic |
| 41 | MDS-RS-MLD | 2 | Substitution | SF3B1 | c.2098A>G |  | oncogenic |
| 42 | MDS with 5q- | 2 | Substitution | SF3B1 | c.2387A>G |  |  |
| 42 | MDS with 5q- | 20 | Substitution | ASXL1 | c.2387G>A |  | oncogenic |
| 43 | MDS/MPN-RS-T | 12 | Substitution | SH2B3 | c.514A>T | p.(Lys181*) |  |
| 43 | MDS/MPN-RS-T | X | Substitution | ZRSR2 | c.94C>T | p.(Gln32*) |  |
| 44 | MDS-RS-MLD | 2 | Substitution | SF3B1 | c.2098A>G | p.Lys700Glu | oncogenic |
| MDS-RS-MLD myelodysplastic syndrome with ring sideroblasts multilineage dysplasia; MDS-MLD myelodysplastic syndrome single lineage dysplasia; MDS-SLD myelodysplastic syndrome single lineage dysplasia; MDS-RS-SLD myelodysplastic syndrome with ring sideroblasts single lineage dysplasia; MDS-EB1 myelodysplastic syndrome with excess blasts-1; MDS/MPN-RS-T myelodysplastic syndrome/myeloproliferative neoplasm with ring sideroblasts and thrombocytosis. | | | | | | | |

**Supplementary table 5. Laboratory features according to the presence of somatic mutations.**

|  | **Mutated** | **Unmutated** | **p** |
| --- | --- | --- | --- |
| **SF3B1** |  |  |  |
| **Mean BM cellularity %** | 55+19 | 40,8+15 | 0,01 |
| **Mean PLT +SD x10^9/L** | 263+129 | 163+96 | 0,01 |
| **Mean ANC +SD x10^9/L** | 3.1+1.7 | 2+11 | 0,01 |
| **DNMT3A** |  |  | **p** |
| **Mean creatinine +SD (mg/dL)** | 0,6+0,15 | 0,9+0,23 | 0,05 |
| **Mean Hb +SD (g/dL)** | 11+1,9 | 9,9+1,5 | 0,06 |
| **U2AF1** |  |  | **p** |
| **Mean BM cellularity %** | 37+5 | 47+18 | 0,03 |
| **Mean LDH +SD (U/L)** | 146+23 | 207+40 | 0,005 |
| **ASXL1** |  |  | p |
| **Mean ANC +SD x10^9/L** | 1.18+0.6 | 2.5+1.4 | 0,04 |
| **Mean endogenous EPO U/L** | 166+146 | 84+57 | 0,03 |
| **JAK2** |  |  | p |
| **Mean Hb +SD (g/dL)** | 12+2 | 10+1,6 | 0,05 |
| **Mean PLT +SD x10^9/L** | 397+296 | 187+164 | 0,01 |
|  | **Mutated** | **Unmutated** | **p** |
| **Splicing genes** |  |  |  |
| **Mean age, years** | 74+6 | 64+12 | 0,01 |
| **Mean PLT +SD x10^9/L** | 225+126 | 153+86 | 0,05 |
| **Mean ANC +SD x10^9/L** | 2.5+1.5 | 1.8+0.9 | 0,015 |
| **Mean ALC +SD x10^9/L** | 1.6+ 0.4 | 1.3+0.4 | 0,04 |
| **DNA methylation genes** |  |  | p |
| **Mean ANC +SD x10^9/L** | 1.6+0.9 | 2.8+1.5 | 0,01 |
| **Mean BM cellularity %** | 39+9 | 49+20 | 0,05 |
| **Chromatin modifiers genes** |  |  | p |
| **Mean ANC +SD x10^9/L** | 1.1+0.6 | 2.5+1.4 | 0,04 |
| **Mean endogenous EPO U/L** | 166+146 | 84+57 | 0,03 |
| **Transcriptional factors genes** |  |  | p |
| **Mean endogenous EPO U/L** | 183+123 | 85+64 | 0,02 |

PLT platelets; ANC absolute neutrophile count; ALC absolute lymphocyte count; BM bone marrow; EPO erythropoietin; Hb hemoglobin; LDH lactate dehydrogenase; SD standard deviation.

SF3B1 mutation significantly correlated with greater levels of PLT and ANC, and higher cellularity. The presence of DNMT3A mutation was associated with higher Hb levels and lower creatinine. U2AF1 mutation was related to lower cellularity and lower LDH. Patients with ASXL1 mutation had lower ANC and higher levels of endogenous EPO. JAK2 mutation was related to higher Hb and PLT values. By grouping mutations into functional categories, splicing alterations (N=34) correlated with older age, higher ANC, PLT, and ALC, and lower LDH. DNA methylation mutations (N=19) were associated with lower ANC and cellularity. Patients with chromatin modifiers mutations (N=6) showed lower ANCs and higher endogenous EPO. The same was observed for patients with mutations in transcription factors (N=3).

**Supplementary table 6. Patients with low-risk myelodysplastic syndromes classified according to a personalized prediction model including molecular features.**

|  | **Very low risk**  **(N=31)** | **Low risk**  **(N=22)** | **Intermediate risk (N=3)** |
| --- | --- | --- | --- |
| **24-month probability of survival %** | 40 (32-50) | 41 (28-54) | 44 (42-47) |
| **24-month probability of leukemic evolution %** | 12.5 (6-23) | 14 (6-44) | 12 (11-13) |
| **60-month probability of survival %** | 16 (9-25) | 16 (7-24) | 18 (18-24) |
| **60-month probability of leukemic evolution %** | 20 (9-71) | 19 (12-54) | 18 (17-20) |
